# Clonal Hematopoiesis and Gut Microbiota-Derived TMAO as Candidate Amplifiers of Cardiovascular Inflammation: The CHIDT Hypothesis

**DOI:** 10.3390/antiox15060781

**Published:** 2026-06-22

**Authors:** Eugenio Caradonna, Fulvio Ferrara, Lucy Costantino, Fortuna Iannuzzo, Nicola Testa, Luca Giordano, Alice Faversani, Carlo Setacci, Ettore Novellino, Emilio Vanoli

**Affiliations:** 1 Integrated Laboratory Medicine Services, Centro Diagnostico Italiano S.p.A., 20011 Milan, Italy; 2 Department of Experimental and Clinical Pathology, IRCCS Istituto Auxologico Italiano, 20149 Milan, Italy; f.ferrara@auxologico.it; 3 Laboratory of Medical Genetics, Centro Diagnostico Italiano S.p.A., 20011 Milan, Italy; lucy.costantino@cdi.it (L.C.); luca.giordano@cdi.it (L.G.); alice.faversani@cdi.it (A.F.); 4 Department of Pharmacy, University of Chieti-Pescara G. D’Annunzio, 66100 Chieti, Italy; 5 Department of Cardiovascular Sciences, Fondazione Policlinico Universitario “A. Gemelli” IRCCS, 00168 Rome, Italy; nicola.testa@policlinicogemelli.it; 6 Vascular Surgery Department, Postgraduate School in Vascular Surgery, University of Siena, 53100 Siena, Italy; carlo.setacci@unisi.it; 7 Department of Medicine and Surgery, Catholic University of the Sacred Heart, 00168 Rome, Italy; ettore.novellino@unicatt.it; 8 School of Nursing, University of Pavia, 27100 Pavia, Italy; emilio.vanoli@unipv.it

**Keywords:** clonal hematopoiesis, TET2, DNMT3A, trimethylamine N-oxide, gut dysbiosis, NLRP3 inflammasome, pyroptosis, cardiovascular risk, atherosclerosis, nutraceutical

## Abstract

Clonal hematopoiesis of indeterminate potential (CHIP) and the gut microbiota-derived metabolite trimethylamine N-oxide (TMAO) are both linked to NLRP3-mediated cardiovascular inflammation, but their interaction has not previously been explored. This work proposes the CHIDT axis (clonal hematopoiesis–dysbiosis–TMAO), a feed-forward mechanism in which TET2 loss-of-function CHIP- and TMAO-generating Gram-negative gut dysbiosis mutually enhance cardiovascular risk. The model proceeds in three nodes. CHIP-associated intestinal immune dysregulation promotes luminal expansion of Gammaproteobacteria, which produce both trimethylamine via CntA/CntB-mediated L-carnitine oxidation and ADP-heptose as an obligate LPS biosynthetic intermediate. TMAO amplifies NLRP3 inflammasome activation through the SIRT3 → SOD2 → mtROS pathway. The evidence base of the CHIDT model is strongest for TET2-CHIP; the proposed extension to DNMT3A-CHIP rests on indirect, associative data and requires dedicated experimental confirmation before it can be considered established. TXNIP cascade, with predicted disproportionate potency in macrophages epigenetically primed by TET2 haploinsufficiency. High concentrations of TMAO have also been shown to suppress TET2 expression in endothelial cells through CYTB promoter hypermethylation, inducing NLRP3–GSDMD-dependent pyroptosis, although it remains unclear whether physiological TMAO levels can trigger this effect. Concurrently, ADP-heptose activates the ALPK1–TIFA–NF-κB pathway in bone marrow progenitors, favoring the expansion of mutant hematopoietic stem and progenitor cells. The model identifies three potential therapeutic strategies: NLRP3 inhibition, microbial TMA lyase inhibition, and microbiome-targeted reduction in Gram-negative bacteria. None has been tested in CHIP carriers stratified by plasma TMAO. Further studies in preclinical models and human cohorts integrating CHIP genotyping and TMAO quantification are needed to validate the CHIDT axis as a target for precision cardiovascular prevention.

## 1. Introduction

A substantial fraction of cardiovascular events arises in individuals with well-controlled traditional risk factors. The inflammatory burden that persists despite optimal pharmacological management of dyslipidemia, hypertension, and dysglycemia has driven sustained investigation into unconventional atherogenic mechanisms [1,2]. Clonal hematopoiesis of indeterminate potential (CHIP) and the gut microbiota-derived metabolite trimethylamine N-oxide (TMAO) have independently emerged as candidates in this process, as each one affects cardiovascular outcomes. Yet, mechanistic interaction among them has not been systematically examined to date.

CHIP is defined by the clonal expansion of hematopoietic stem cells harboring somatic driver mutations, most commonly in *TET2*, *DNMT3A*, *ASXL1*, and *JAK2*, at a variant allele frequency ≥2% in peripheral blood, without cytopenias or overt hematological malignancy [3,4]. The condition affects approximately 10–15% of individuals over the age of 70 and independently confers roughly a twofold increase in atherosclerotic cardiovascular disease risk. The TMAO pathway runs on a parallel but convergent track: dietary choline and L-carnitine are catabolized by gut bacteria to trimethylamine (TMA), which hepatic FMO3 then oxidizes to TMAO [5]. The metabolite drives NLRP3-dependent vascular inflammation through mechanisms that overlap substantially with those activated by somatic TET2 loss in hematopoietic cells, an overlap that motivates the analysis and the hypothesis presented here [6].

We describe a proposed CHIDT axis linking CHIP and TMAO in a cycle of mutual reinforcement. The model is grounded primarily in TET2-CHIP, for which the mechanistic evidence is most complete. DNMT3A-CHIP is considered separately as a secondary and mechanistically distinct extension. Throughout, we state explicitly which steps rest on established experimental evidence, which represent plausible inference from indirect data, and which remain untested.

## 2. CHIP: A Somatic Driver of Cardiovascular Inflammation

### 2.1. Epidemiology and Mechanism

CHIP’s cardiovascular relevance emerged from large-scale exome sequencing studies demonstrating that age-acquired somatic mutations in leukemia driver genes independently predict coronary artery disease and all-cause mortality in individuals without hematological malignancy [3]. Mutation-specific risk is not uniform. *TET2* and *JAK2* mutations carry the highest cardiovascular hazard; *DNMT3A* mutations are associated with a more attenuated vascular phenotype. Clone size exerts an independent, dose-dependent effect: as the fraction of hyperinflammatory circulating myeloid cells expands, vascular homeostasis erodes progressively [7].

In UK Biobank analyses, *TET2*-CHIP with variant allele fraction ≥10% independently predicted adverse outcomes at ten-year follow-up, with an adjusted hazard ratio reaching 1.89 [7]. The underlying mechanism is well characterized. TET2 loss epigenetically derepresses inflammatory gene programs governing NLRP3 assembly and IL-1β processing, establishing a cell-autonomous hyperinflammatory state. Fuster et al. showed that partial reconstitution of *Ldlr*^−^/^−^ mice with *Tet2*-deficient hematopoietic cells markedly increased atherosclerotic plaque burden, and that this phenotype was selectively reversed by NLRP3 inhibition [8]. Single-cell transcriptomic profiling of circulating monocytes from patients with established cardiovascular disease confirmed the same transcriptionally primed state in human tissue [9]. CANTOS provided clinical precedent: IL-1β blockade with canakinumab reduced cardiovascular events independently of LDL-cholesterol levels [10].

The mechanistic distinction between *TET2* and *DNMT3A* mutations is consequential for the CHIDT framework. *TET2*-mutant macrophages are cell-autonomously hyperinflammatory. *DNMT3A* mutations, by contrast, confer a self-renewal advantage to mutant hematopoietic stem and progenitor cells (HSPCs) by disrupting polycomb-target methylation, without amplifying per-cell cytokine output to the same degree. The CHIDT model is therefore mechanistically anchored in *TET2* biology; extension to *DNMT3A*-CHIP requires dedicated experimental work before it can be treated as anything more than a working hypothesis.

Within the *TET2*-CHIP arm, the NLRP3 effector logic is well supported. Polizio et al. demonstrated that *Tet2*-deficient hematopoietic cell transfer sensitizes mice to angiotensin II-induced hypertension through renal macrophage infiltration and NLRP3 activation, a phenotype fully reversed by the selective NLRP3 inhibitor MCC950 [11]. *TET2*-CHIP is therefore one of the few human genetic cardiovascular risk factors for which a directly tractable inflammasome pharmacology has been demonstrated.

### 2.2. DNMT3A-CHIP: A Secondary and More Speculative Extension

No published study has directly demonstrated that TMAO promotes the expansion of DNMT3A-mutant hematopoietic clones, and the biological mechanism through which TMAO might interact with DNMT3A-mutant cells differs substantially from the TET2-CHIP effector pathway described above. The following discussion retains DNMT3A-CHIP within the CHIDT framework solely because the overlapping gut microbial ecology of ADP-heptose and TMAO production is mechanistically pertinent to the model as a whole; all DNMT3A-specific propositions should be read as exploratory hypotheses, not established interactions.

Incorporating *DNMT3A*-CHIP into the CHIDT framework requires explicit acknowledgment of its different biology. TMAO may influence *DNMT3A*-CHIP not as a direct inflammasome trigger but as a dual epigenetic cofactor. Population-scale epigenome-wide association studies have identified associations between TMAO-related choline metabolites and DNA methylation changes at NF-κB and TNF signaling loci [12]. The microbially derived ADP-heptose—produced by all Gram-negative bacteria—drives *DNMT3A*-mutant HSPC expansion through ALPK1–TIFA–NF-κB signaling [13]. Both observations are suggestive; neither constitutes direct evidence for TMAO-driven expansion of *DNMT3A*-mutant clones. We retain *DNMT3A*-CHIP in this discussion because the overlapping gut microbial ecology of ADP-heptose and TMAO production is pertinent. However, the evidentiary basis is substantially weaker than for the *TET2* arm.

## 3. TMAO and Gut Dysbiosis: From Microbial Ecology to Vascular Injury

### 3.1. Biosynthesis and Clinical Associations

Sustained TMAO production requires two conditions: a gut microbial community with sufficient TMA-lyase activity, and a functionally competent hepatic FMO3, which is approximately tenfold more active than FMO1 in TMAO synthesis [14].

Multiple bacterial enzymatic routes contribute: the CutC/CutD complex cleaves choline; CntA/CntB and YeaW/YeaX oxidoreductases process L-carnitine and betaine, respectively. The periodontal pathobiont *Porphyromonas gingivalis* directly upregulates hepatic FMO3 expression [15,16], linking oral microbiome dysbiosis to elevated systemic TMAO. FMO3 is also expressed in vascular endothelial cells, macrophages, and smooth muscle cells, raising the possibility of local vascular TMAO synthesis [17,18]. Testosterone-mediated suppression of FMO3 transcription accounts for the consistently lower fasting TMAO concentrations observed in males. A dose–response meta-analysis in 31,230 participants confirmed a positive, non-linear association between plasma TMAO and all-cause mortality; individuals in the highest exposure category carried a 47% increase in mortality hazard [19]. At fasting concentrations of 0.11–6.45 μmol/L, as encountered in most omnivorous Western populations, TMAO behaves as a protein-stabilizing osmolyte with potential cytoprotective properties [20]. In the CHIDT framework, cardiovascular risk is concentrated at the upper range of exposure. Plasma TMAO also correlates with carotid intima-media thickness (r = 0.26; *p* < 0.0001) independently of established risk factors [21].

### 3.2. Mechanisms of TMAO-Induced Vascular Injury

The initiating event in TMAO-induced vascular toxicity is mitochondrial. TMAO inhibits sirtuin-3 (SIRT3), impairing SIRT3–SOD2-mediated activation of superoxide dismutase 2 (SOD2). The resulting mitochondrial ROS (mtROS) accumulation dissociates thioredoxin-interacting protein (TXNIP) from thioredoxin, liberating TXNIP to bind NLRP3 and license inflammasome assembly. This SIRT3→SOD2→mtROS→TXNIP–NLRP3 cascade has been established in HUVEC cultures and ApoE^−^/^−^ aortic tissue [6]. Further injury mechanisms include MAPK/JNK-mediated upregulation of scavenger receptors CD36 and SR-A1 with impaired CYP7A1/CYP27A1-dependent reverse cholesterol transport [22,23], eNOS inhibition with uncoupled superoxide generation [24], and JAK2–STAT3-driven cardiac fibrosis [25]. Clinically, elevated TMAO has also been associated with adverse outcomes in Norwegian heart failure cohorts [26].

In white adipose tissue, age-dependent upregulation of adipocyte FMO3 drives local TMAO accumulation; the metabolite binds directly to the inflammasome adaptor ASC, promoting caspase-1 activation and IL-1β production and connecting TMAO to the senescence-associated inflammatory phenotype of aging adipose tissue [17]. In patients with prior ischemic stroke, plasma TMAO correlated strongly with the CD14^++^CD16^+^ intermediate monocyte fraction (r = 0.70; *p* < 0.01) [27], a subset characterized by enhanced adhesion to activated endothelium and preferential secretion of TNF-α, IL-6, and MMP-9. At the macrophage level, TMAO activates the PERK branch of the unfolded protein response, driving CREB/ATF4 reprogramming toward a pro-inflammatory glycolytic phenotype consistent with trained innate immunity [18].

### 3.3. Gut Dysbiosis as the Upstream Driver

Three enzymatic routes contribute substantially to luminal TMA generation and deserve separate consideration, as their taxonomic distributions carry distinct implications for the CHIDT model. The CutC/CutD choline TMA-lyase, carried predominantly by Gram-positive Firmicutes, including Lachnoclostridium, Clostridium XIVa, and Eubacterium spp., is quantitatively the dominant pathway in Western diets with high phosphatidylcholine intake. The CntA/CntB (also designated YeaW/YeaX) carnitine oxygenase, concentrated almost exclusively in Gammaproteobacteria, converts dietary L-carnitine to TMA. The gbu gene cluster mediates betaine-derived TMA production across Clostridiales. These routes are not mutually exclusive and contribute in proportion to substrate availability and community composition. The emphasis that follows on Gammaproteobacteria and CntA/CntB does not imply that CutC/CutD-mediated TMAO generation is less important; rather, it reflects a specific structural argument central to the CHIDT model: a single age-related Gammaproteobacterial bloom simultaneously generates both molecular outputs of the dysbiotic arm—TMAO via CntA/CntB and ADP-heptose via obligate LPS biosynthesis—as dual products of the same ecological shift rather than independent perturbations. The relative contribution of CutC/CutD-producing Firmicutes to the CHIDT axis awaits metagenomics characterization in CHIP carrier cohorts.

The TMA-producing capacity of the gut microbiome, rather than dietary substrate availability alone, is the principal determinant of inter-individual TMAO variability. The dysbiotic ecology associated with chronically elevated TMAO, characterized by depletion of butyrate-producing commensals, increased intestinal permeability, and LPS translocation, superimposes a TLR4-mediated inflammatory stimulus on the direct pro-atherogenic effects of TMAO itself [28,29]. A taxonomically important observation deepens the mechanistic coherence of this picture. TMA production from L-carnitine is catalyzed by the CntA/CntB (also designated YeaW/YeaX) carnitine oxygenase pathway, which is concentrated almost exclusively in Gammaproteobacteria, including *Escherichia coli*, *Klebsiella pneumoniae*, *Acinetobacter baumannii*, and *Proteus mirabilis*, with *cntA* amplicons showing ~99% sequence identity to Gammaproteobacterial references in large-scale fecal metagenomes [30]. Integrated metagenomics across 2134 individuals confirmed that all 13 strains with detectable *cntA* sequences belonged to Proteobacteria [31,32]. The same Gammaproteobacterial clade also generates ADP-heptose as an obligate LPS biosynthetic intermediate. Age-related Gammaproteobacterial expansion therefore simultaneously produces both molecular signals central to the CHIDT axis: TMAO for the *TET2*-CHIP arm and ADP-heptose for the *DNMT3A*-CHIP arm, as dual outputs of a single dysbiotic shift, not independent perturbations.

## 4. TET2 Suppression as a Candidate Molecular Nexus

The most direct mechanistic argument for a CHIP × TMAO interaction derives from Xia et al., who showed that TMAO suppresses *TET2* expression in vascular endothelial cells (VECs) through CYTB promoter hypermethylation, triggering NLRP3-dependent pyroptosis [33]. The pathway is mechanistically compelling: reduced TET2 expression promotes methylation of the cytochrome b (*CYTB*) gene promoter, decreasing CYTB transcription, impairing mitochondrial function, accumulating mtROS, and sequentially activating NLRP3, caspase-1, gasdermin D, and IL-1β/IL-18 release. The critical caveat is that these results were obtained at TMAO concentrations (600 μmol/L for 24 h in the culture medium) approximately two orders of magnitude above physiological fasting plasma levels in healthy individuals. The finding is best interpreted as proof of concept for a TET2→NLRP3 cascade in the vascular compartment, not as direct translational evidence.

Three features of the experimental context support the pathophysiological plausibility of this mechanism at lower, clinically relevant concentrations, while simultaneously defining where evidence ends and extrapolation begins. Patients with end-stage renal disease sustain circulating TMAO concentrations of 50–100 μmol/L or higher due to impaired tubular secretion, a population carrying markedly elevated cardiovascular risk. Intracellular TMAO concentrations in endothelial cells may substantially exceed plasma values, making direct comparisons with fasting plasma measurements potentially misleading. Most consequential for the CHIP context, *TET2* haploinsufficiency may lower the concentration threshold at which exogenous TMAO induces functional TET2 insufficiency, because the remaining wild-type allele operates with reduced redundancy. The 600 μmol/L threshold of Xia et al. is not an outlier. Cardiomyocytes show TGF-β1/Smad3 hypertrophic activation at low micromolar TMAO concentrations [34]. Colonic epithelial cells lose ATG16L1-dependent autophagy and disinhibit NLRP3 at 200–300 μmol/L, a range reached in end-stage renal disease and after acute choline loading [35]. Endothelial SIRT3–SOD2–mtROS-dependent NLRP3 activation has been described from 150 μmol/L upward [6]. The 600 μmol/L figure sits at the top of this range, not outside it. Whether the threshold shifts further downward in *Tet2*^+^/^−^ myeloid cells, where inflammatory gene programs are already epigenetically disinhibited, is an open question. The experiment is not complicated: *Tet2*^+^/^−^ macrophages, TMAO at 1–50 μmol/L, NLRP3 assembly, and IL-1β output measured. Until those data exist, this node of the CHIDT cycle is mechanistically grounded but experimentally unconfirmed.

A parallel post-translational mechanism may compound *TET2* suppression independently of transcriptional repression. SIRT1 deacetylates the catalytic domain of TET2 at lysine residues K1472, K1473, and K1478, a modification required for full dioxygenase processivity [36]. TMAO-driven NAD^+^ depletion, a predictable consequence of sustained mitochondrial oxidative stress, would reduce SIRT1 catalytic activity, leaving TET2 in a hyperacetylated, catalytically impaired state, a post-translational inactivation that phenocopies TET2 protein loss without altering transcript abundance. This mechanism was established in MDS hematopoietic progenitors; its relevance to CHIP-associated cardiovascular contexts remains untested. As an Fe^2+^- and α-ketoglutarate (α-KG)-dependent dioxygenase, TET2 catalytic output is therefore not a fixed cellular property but a dynamic function of metabolic and redox cofactor availability: α-KG determines substrate access, while ascorbate maintains catalytic iron in the Fe^2+^ state required for dioxygenase processivity. In the CHIDT context, TMAO-driven mitochondrial dysfunction may simultaneously deplete both cofactors, reducing α-KG through TCA cycle perturbation and consuming ascorbate through oxidative stress, compounding transcriptional TET2 suppression with post-translational catalytic insufficiency. Whether this convergent mechanism operates at clinically encountered TMAO concentrations in TET2-haploinsufficient cells warrants dedicated investigations.

## 5. The CHIDT Model

**Figure 1:**
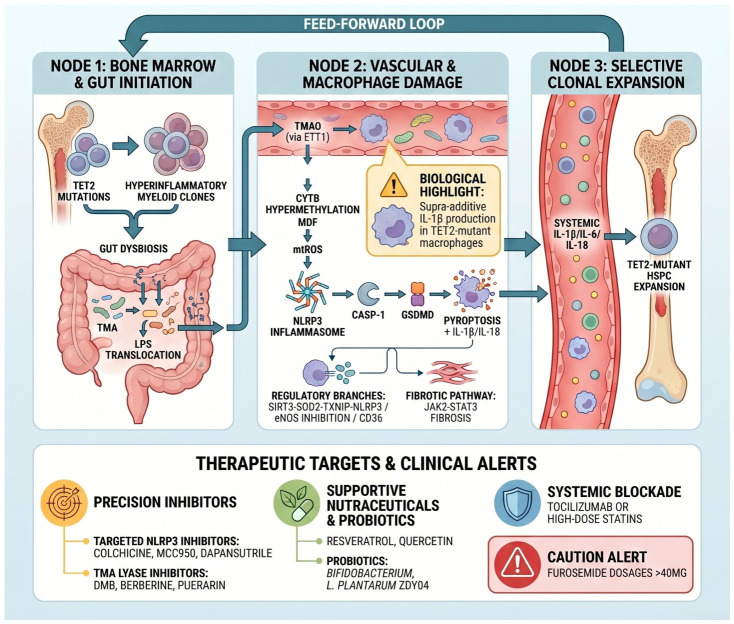
**The CHIDT (clonal hematopoiesis–dysbiosis–TMAO) feed-forward inflammation cycle.** This image illustrates the **CHIDT axis**, a self-reinforcing pathogenic loop where genetic instability, gut microbial imbalance, and metabolic signaling converge to accelerate cardiovascular and renal diseases. **Node 1: Clonal Hematopoiesis and Gut Dysbiosis.** Somatic mutations in hematopoietic stem and progenitor cells (HSPCs)—primarily **TET2**—give rise to hyper-inflammatory myeloid clones. These cells disrupt intestinal immune surveillance, promoting **gut dysbiosis** characterized by an expansion of TMA-producing Gram-negative bacteria and the translocation of lipopolysaccharides (**LPS**) into the systemic circulation; **Node 2: TMAO-Mediated Vascular and Macrophage Damage.** Microbial trimethylamine (TMA) is oxidized by the liver into **trimethylamine N-oxide (TMAO)**. TMAO enters vascular endothelial cells (VECs) via the endothelial TMAO transporter 1 (ETT1) [37], where it suppresses **TET2 expression** and induces **CYTB promoter hypermethylation**, leading to **mitochondrial dysfunction (MDF)** and the accumulation of reactive oxygen species (**mtROS**). This triggers the **NLRP3 inflammasome** cascade (Caspase-1 and Gasdermin D), resulting in **pyroptosis** and the release of **IL-1β and IL-18**. Notably, in TET2-mutant macrophages, this signal generates a **supra-additive IL-1β release**, intensifying vascular injury and **JAK2–STAT3-mediated fibrosis; Node 3: Selective Clonal Expansion.** The resulting systemic inflammatory milieu, rich in **IL-1β, IL-6, and IL-18**, creates a selective growth advantage for **TET2-mutant HSPCs**. This drives further expansion of the mutant clones, which in turn exacerbates intestinal immune dysregulation (Node 1), thereby closing the **pathogenic feed-forward loop. Therapeutic Interventions:** Strategic targets to break this cycle include NLRP3/IL-1β inhibitors: colchicine, MCC950, and canakinumab, which have shown efficacy in reducing cardiovascular events particularly in CHIP carriers; microbiome and TMAO modulation: TMA lyase inhibitors (DMB, berberine), probiotics (Bifidobacterium), and plant-based diets; nutraceuticals and statins: resveratrol, quercetin, and high-dose statins for their pleiotropic anti-inflammatory and mitochondrial protective effects. **Clinical Caution:** High doses of loop diuretics (e.g., furosemide >40 mg) may paradoxically increase systemic TMAO levels by impairing its renal excretion, potentially fueling the CHIDT cycle.

The CHIDT model is presented in Figure 1. The figure provides a schematic representation of the model and illustrates its main components and their interrelationships, facilitating a clearer understanding of the conceptual framework described in the manuscript. Node 1: Clonal Hematopoiesis and Intestinal Immune Dysregulation. Somatic TET2 mutations generate hyperinflammatory myeloid clones that impair intestinal barrier integrity and alter lamina propria immune surveillance, promoting luminal expansion of TMA-producing Gram-negative bacteria and LPS translocation. This circuit has been demonstrated in Tet2^−^/^−^ mice and reversed by anti-IL-6 treatment, antibiotics, or germ-free rederivation [37]; whether it generates TMA-producing dysbiosis proportional to clone size in humans remains to be shown.

Node 2: TMAO-Mediated Vascular and Macrophage Injury. Gut-derived TMA is hepatically oxidized to TMAO, which enters VECs, suppresses TET2 via CYTB promoter hypermethylation, and activates the NLRP3–caspase-1–GSDMD axis. This pathway is established at supraphysiological TMAO concentrations (~600 μmol/L) [34]; whether it operates at clinically encountered levels in TET2-haploinsufficient cells is the central experimental question of the model. In TET2-mutant macrophages, supra-additive IL-1β release is predicted but not yet demonstrated.

Node 3: Selective Clonal Expansion. The resulting IL-1β/IL-6/IL-18 milieu selectively favors TET2-mutant HSPC expansion, increasing clone size and intensifying intestinal immune dysregulation (Node 1), closing the feed-forward loop. ADP-heptose from Gram-negative LPS biosynthesis provides an independent ALPK1–TIFA–NF-κB clonal selection signal, established in vivo by Agarwal et al. [14]. Table 1 summarizes the strength of evidence for each node of the CHIDT model and the main remaining knowledge gaps.

**Table 1:** Strength of evidence supporting each node of the CHIDT model and the principal remaining knowledge gaps. Evidence levels are graded as established, indirect, or speculative.

CHIDT Step	Proposed Interaction	Evidence Level	Evidence Source	Key Knowledge Gap
Node 1	CHIP (TET2) myeloid cells impair the gut barrier → TMA-producing Gram-negative dysbiosis and LPS translocation	Indirect; established in mice	Animal (Tet2^−^/^−^; Meisel et al.)	Whether dysbiosis scales with variant allele fraction in humans
Dysbiosis	Age-related Gammaproteobacterial bloom yields TMAO (CntA/CntB) and ADP-heptose (LPS) as dual outputs	Established microbial ecology; indirect for the CHIP link	Human metagenomics (2134 subjects)	Causal link between CHIP genotype and TMA-producer abundance
Node 2 (bridge)	TMAO suppresses TET2 via CYTB promoter hypermethylation → NLRP3–GSDMD pyroptosis	Established in vitro but only at supraphysiological ~600 μmol/L	In vitro/animal (Xia et al.)	Whether it operates at clinically encountered TMAO levels in TET2-haploinsufficient cells
Node 2 (synergy)	Supra-additive NLRP3/IL-1β output in TET2-mutant macrophages exposed to TMAO	Speculative/predicted	Not yet demonstrated	The single most tractable untested in vitro experiment
Node 3	ADP-heptose → ALPK1–TIFA–NF-κB → selective clonal expansion	Established for ADP-heptose/ALPK1; indirect within CHIDT	Animal (Agarwal et al.)	Relative contribution versus the TMAO–NLRP3 pathway
Effector axis	TET2-CHIP → NLRP3-driven atherogenesis/hypertension; reversed by NLRP3 or IL-1β inhibition	Established (animal) with clinical precedent (CANTOS)	Animal (Fuster; Polizio) + human (CANTOS)	Not yet tested in CHIP carriers stratified by plasma TMAO
DNMT3A arm	TMAO acts as an epigenetic cofactor for DNMT3A-mutant clones	Speculative	Associative (epigenome-wide studies)	No demonstration of TMAO-driven DNMT3A clonal expansion

### 5.1. Node 1: CHIP → Altered Intestinal Immune Surveillance

*TET2*-deficient macrophages and monocytes circulate in a hyperinflammatory state through all vascular beds, including the intestinal lamina propria. Direct experimental evidence that this disrupts microbial ecology comes from Meisel et al., who demonstrated in *Tet2*^−^/^−^ mice that TET2-deficient pro-inflammatory myeloid cells impair intestinal barrier integrity and generate systemic bacterially driven inflammation that further amplifies *TET2*-mutant clone expansion; this cycle was fully reversed by anti-IL-6 treatment, antibiotic administration, or germ-free rederivation [38,39]. The systemic IL-1β and IL-6 excess characteristic of *TET2*-CHIP likely contributes to tight junction disruption and LPS translocation [10,28], compounding the TMAO-independent vascular inflammatory burden. Whether this gut–immune–CHIP circuit generates specifically TMA-producing dysbiosis in proportion to variant allele fraction has not been measured in humans.

### 5.2. Node 2: Dysbiosis → Elevated TMAO → NLRP3 Activation

**Figure 2:**
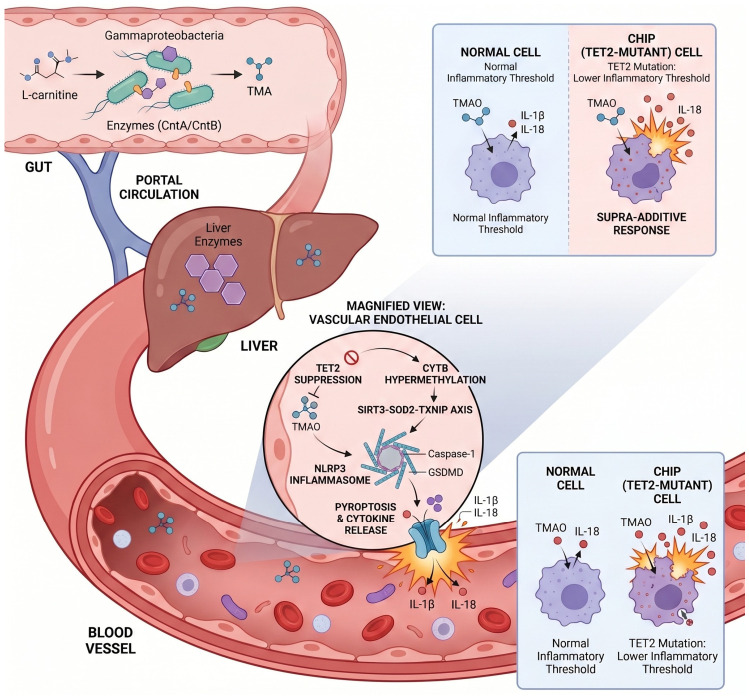
**Molecular mechanisms of TMAO-induced vascular and cardiac injury in the context of clonal hematopoiesis.** This infographic illustrates the molecular cascade triggered by the gut microbiota-derived metabolite, **trimethylamine N-oxide (TMAO)**, and its synergistic interaction with **clonal hematopoiesis of indeterminate potential (CHIP)**: **(1) gut–liver axis and production:** dietary precursors such as choline and L-carnitine are metabolized by specific gut bacteria (e.g., *Gammaproteobacteria*) into trimethylamine (TMA), which is subsequently oxidized to TMAO by hepatic flavin monooxygenases (FMOs); **(2) cellular entry and epigenetic modification:** TMAO enters vascular endothelial cells (VECs) and macrophages, where it facilitates **CYTB promoter hypermethylation** and directly suppresses **TET2 expression**; **(3) mitochondrial dysfunction (MDF) and oxidative stress:** these epigenetic changes lead to mitochondrial impairment, characterized by the inhibition of pyruvate dehydrogenase and the accumulation of **mitochondrial reactive oxygen species (mtROS)**; **(4) inflammasome activation and pyroptosis:** the resulting oxidative stress triggers the **SIRT3–SOD2–TXNIP–NLRP3 axis**, leading to the assembly of the **NLRP3 inflammasome** (this process activates **caspase-1**, which cleaves gasdermin D (GSDMD), culminating in **pyroptosis** and the release of highly pro-inflammatory cytokines, **IL-1β and IL-18)**; **(5) synergy with CHIP clones:** in individuals with CHIP, particularly those with **TET2 mutations**, myeloid cells are already epigenetically primed for inflammation. Exposure to TMAO in these mutant macrophages results in a **“supra-additive” inflammatory response**, disproportionately increasing IL-1β secretion and accelerating **atherogenesis, cardiac fibrosis, and heart failure**. **Therapeutic targets:** potential interventions include **NLRP3 inhibitors** (e.g., colchicine), **TMA lyase blockers**, and the modulation of the gut microbiota through **plant-based diets** or probiotics to reduce systemic TMAO levels.

The Gammaproteobacterial ecological shift predicted by Node 1 drives sustained TMA production via CntA/CntB-mediated L-carnitine oxidation. TMAO enters VECs and, based on current in vitro evidence obtained at supraphysiological concentrations, may suppress *TET2*, hypermethylate the *CYTB* promoter, and trigger NLRP3–caspase-1–GSDMD–IL-1β/IL-18 pyroptosis [33]. The SIRT3→SOD2→TXNIP–NLRP3 axis contributes independently [6]. In *TET2*-mutant macrophages derived from expanded CHIP clones, with NLRP3 already deubiquitylated and primed at a lower activation threshold, this TMAO-derived inflammasome signal is predicted to generate a disproportionate IL-1β response. Supra-additive NLRP3 activation in *Tet2*-deficient macrophages exposed to pathophysiologically relevant TMAO concentrations has not been demonstrated; this is the most critical and immediately tractable experimental test of the model (Figure 2).

### 5.3. Node 3: Amplified Inflammation → Clonal Expansion

The heightened systemic IL-1β, IL-6, and IL-18 milieu resulting from convergent NLRP3 hyperactivation provides a selective growth advantage to *TET2*-mutant HSPCs within the bone marrow niche, increasing clone size and the circulating fraction of hyperinflammatory myeloid cells. The bone marrow milieu is not sustained by NLRP3-derived cytokines alone. Gram-negative bacterial products translocating across the disrupted intestinal barrier—principally ADP-heptose, the LPS biosynthetic intermediate produced by all Gammaproteobacteria—reach the bone marrow via portal and systemic circulation, where they activate ALPK1–TIFA–NF-κB in resident myeloid progenitors. The ADP-heptose–ALPK1 axis may be the more direct upstream driver of clonal selection, acting independently from the TMAO–NLRP3 pathway. TMAO is therefore better understood as a potent amplifier of the established CHIP state than a primary inducer of clonal emergence: its convergent NLRP3 activation in *TET2*-deficient macrophages raises circulating IL-1β and IL-18, sustaining the cytokine milieu that selects for mutant clone growth [3,40]. Larger clones intensify intestinal immune dysregulation (Node 1), closing the amplification loop. Whether TMAO-associated sympathovagal imbalance additionally attenuates cholinergic anti-inflammatory signaling via α7nAChR [41] is an auxiliary hypothesis requiring independent experimental validation before it can be incorporated into the core model.

An important conceptual refinement follows from the evidence reviewed above. The strongest microbiota-linked driver of clonal expansion may not be TMAO itself but the broader Gram-negative dysbiotic milieu from which TMAO arises. In this formulation, Gram-negative expansion and impaired intestinal barrier integrity may provide the primary ecological context for CHIP progression by increasing systemic exposure to pro-inflammatory microbial products, including ADP-heptose, which has recently emerged as a plausible mediator of age-related clonal expansion through ALPK1-dependent signaling [13]. TMAO would then be positioned not as a direct initiator of clonal growth but as a parallel and potentially synergistic amplifier of cardiovascular inflammation, acting through endothelial dysfunction, mitochondrial stress, trained innate immunity, and NLRP3-dependent effector pathways. This distinction matters particularly for *TET2*-CHIP, where myeloid cells are already epigenetically primed toward exaggerated inflammasome activation; within this background, microbiota-derived TMAO may amplify the inflammatory fitness of pre-existing mutant clones even if it does not itself constitute a dominant determinant of clonal emergence. The most defensible formulation of the CHIDT hypothesis is therefore not that TMAO drives CHIP expansion, but that Gram-negative dysbiosis may favor clonal selection while TMAO increases the inflammatory gain of the established CHIP state, together creating conditions for supra-additive cardiovascular injury. Defining the relative contributions of ADP-heptose, TMAO, and other gut-derived inflammatory signals to clone dynamics and vascular risk is a central priority for future mechanistic and translational studies.

**Beyond the NLRP3 inflammasome—complementary innate and adaptive pathways.** Beyond the NLRP3 inflammasome, several additional immune circuits are likely to contribute to CHIP-associated cardiovascular inflammation and warrant integration into future iterations of the CHIDT model. The cytosolic DNA sensor cyclic GMP–AMP synthase–stimulator of interferon genes (cGAS–STING) is directly relevant: TET2 loss provokes DNA damage that activates cGAS–STING in hematopoietic stem and progenitor cells, promoting their self-renewal, while pharmacological or genetic STING inhibition suppresses Tet2-mutant clonal expansion [42]. The same pathway is operative in the vascular compartment, where cGAS–STING signaling drives macrophage pro-inflammatory polarization and atherosclerotic plaque vulnerability [43,44], linking mitochondrial and nuclear DNA stress to the type-I-interferon arm of atherogenesis. In parallel, translocated Gram-negative products engage Toll-like receptor 4 signaling, a long-recognized amplifier of vascular inflammation that operates alongside the ADP-heptose–ALPK1–TIFA axis [45]. Finally, atherosclerosis carries a substantial adaptive-immune and autoimmune component—antigen-specific T-cell responses and regulatory T-cell dysfunction [46]—that may be reshaped by CHIP-driven myeloid signaling and remains essentially unexplored in the CHIDT context.

### 5.4. Testable Predictions

The CHIDT model generates several specific, experimentally falsifiable predictions. *TET2*-CHIP carriers should exhibit elevated plasma TMAO relative to age- and diet-matched non-CHIP controls, after adjustment for renal function and concurrent medication, a question addressable with existing biobank infrastructure combining whole-exome sequencing and targeted metabolomics. Fecal metagenomics in CHIP carrier cohorts should reveal higher *cntA* and LPS core biosynthesis gene (*gmhA*, *hldA*) operon abundance than in non-CHIP controls, correlating with variant allele fraction; simultaneous quantification of plasma TMAO and ADP-heptose would test whether these two gut-derived signals co-elevate in proportion to *TET2* versus *DNMT3A* genotype.

The most critical in vitro experiment is the treatment of *Tet2*-deficient and wild-type murine macrophages with TMAO at 1–50 μmol/L, with measurement of NLRP3 assembly and IL-1β secretion. If haploinsufficient cells respond at concentrations that leave wild-type cells unaffected, the core amplification claim receives direct experimental support. Complementary epigenome profiling (5hmC sequencing) of VECs from *Tet2*-CHIP bone marrow chimeric mice on high-choline diets would test whether dual *TET2* suppression—somatic haploinsufficiency combined with TMAO-driven transcriptional repression—produces a composite hypomethylation phenotype at *CYTB* and inflammatory gene promoters that exceeds either condition independently. The methodological framework established by Agarwal et al. [13] for the ADP-heptose/ALPK1 interaction experiment applies directly to this class of microbial metabolite–CHIP interaction study.

## 6. Therapeutic Implications

No therapeutic strategy has been prospectively tested in CHIP carriers stratified by TMAO burden. The CHIDT framework identifies mechanistically coherent candidates according to their stage of development; however, it does not provide a basis for clinical recommendations in the absence of dedicated validation studies.

### 6.1. NLRP3 Inflammasome Inhibition

NLRP3 blockade is the mechanistically strongest therapeutic candidate in this framework. MCC950 fully reversed *TET2*-CHIP-associated hypertension in murine models [11], and CANTOS established clinical proof of concept for IL-1β inhibition in atherosclerotic cardiovascular disease [10]. Because both TMAO-driven VEC pyroptosis and *TET2*-CHIP converge on the NLRP3–GSDMD–IL-1β/IL-18 effector pathway, an NLRP3 inhibitor addresses both arms simultaneously, a mechanistic rationale absent from standard lipid-lowering therapy. Anti-IL-18 strategies have not been specifically explored in CHIP and may offer complementary benefit at the node where TMAO and *TET2* haploinsufficiency interact. A prospective randomized trial enrolling CHIP carriers stratified by gut TMA-producing capacity and plasma TMAO would provide the definitive clinical test of the CHIDT synergy prediction.

### 6.2. Microbiome-Directed Reduction in TMAO

Non-lethal inhibition of microbial TMA lyase activity represents the most mechanistically specific strategy available. 3,3-Dimethyl-1-butanol (DMB) competitively inhibits the CutC/CutD complex and reduces atherosclerotic lesion area in *ApoE*^−^/^−^ mice on a choline-enriched diet independently of circulating lipid levels [47]. Berberine lowers fecal and plasma TMAO through dual suppression of CutC activity and hepatic FMO3—the latter mediated by its gut-reduced metabolite dihydroberberine—and attenuated carotid plaque progression over four months in a small clinical study of atherosclerotic patients, though sample size limits generalizability [48]. Resveratrol and related polyphenolic compounds reduce TMAO in rodent models through SIRT1/SIRT3 activation and partial restoration of CYP7A1-mediated bile acid flux; human data remain limited to underpowered intervention studies with uncontrolled dietary backgrounds [49]. Several studies have highlighted that polyphenolic phytocomplexes, rich in procyanidins, catechins, epicatechin, resveratrol, and quercetin, are able to significantly modulate plasma levels of TMAO. In particular, these bioactive compounds, commonly found in grape-derived products, exert both antioxidant effects and modulatory activity on the gut microbiota, thereby contributing to the reduction in TMAO production.A clinical study conducted in overweight/obese subjects demonstrated that the administration of a grape pomace polyphenolic extract significantly reduced serum TMAO levels (approximately −78% after 8 weeks). This effect has been attributed both to the modulation of gut microbiota composition and to the direct redox activity of polyphenols, which are able to interfere with oxidative mechanisms involved in TMAO formation [50].

In this context, nutraceutical approaches based on polyphenolic phytocomplexes represent a particularly promising strategy for the modulation of TMAO as a cardiovascular risk biomarker. Selected *Lactobacillus plantarum* and *Bifidobacterium* strains shift gut TMA-producing microbial composition in murine models [51,52]. None of these strategies has been tested in CHIP carriers, and none addresses the upstream dysbiotic ecology that the CHIDT model positions as the primary driver. Their mechanistic plausibility within this framework is noted; clinical translation requires dedicated trials in appropriately genotyped populations. Taira et al. demonstrated in carriers of *germline* TET2 mutations—a clinically distinct entity from somatic CHIP—that oral supplementation with 1 g/day of ascorbate over 12 months enhanced DNA methylation turnover, reduced the proportion of hypermethylated loci at enhancer regions, and partially normalized methylation at hematopoietic transcription factor binding sites; these epigenetic shifts tracked with measurable attenuation of gene expression differences relative to wild-type controls [53]. The mechanistic basis is well established: vitamin C preserves catalytic iron in its reduced Fe^2+^ state, sustaining TET dioxygenase processivity independently of α-KG availability. Whether ascorbate supplementation produces analogous epigenetic restoration in somatic TET2-haploinsufficient hematopoietic clones—where the wild-type allele is intact but functionally insufficient—has not been tested in any cardiovascular CHIP cohort and cannot be inferred from germline data alone. A consequential drug–biomarker interaction warrants explicit consideration in CHIP carriers. Latkovskis et al. demonstrated in a cross-sectional study of 300 cardiovascular patients that loop diuretic use was independently associated with significantly higher plasma TMAO concentrations (log-TMAO 0.510 ± 0.296 vs. 0.336 ± 0.272 in nonusers; *p* = 0.008), and showed in a murine model that furosemide reduces the renal elimination rate of TMAO by 1.9-fold within 30 min of administration, an effect not replicated by probenecid, implicating efflux transporters rather than organic anion transporters as the relevant secretory pathway [54]. In a population where loop diuretics are prescribed for the management of heart failure and volume overload, conditions that track closely with the cardiovascular phenotype associated with CHIP, this pharmacokinetic interaction may systematically elevate circulating TMAO independently of gut dysbiosis, confounding both risk stratification and the interpretation of microbiome-directed interventions. The CHIDT model suggests that the greatest therapeutic benefit in CHIP carriers may require strategies targeting multiple nodes simultaneously: IL-6 receptor blockade to attenuate CHIP-driven intestinal immune dysregulation, TMA lyase inhibition or microbiome-directed TMAO reduction to lower the exogenous inflammasome stimulus, and NLRP3 inhibition to address both CHIP-intrinsic and TMAO-extrinsic components of the amplification cycle. Standard lipid-lowering therapy addresses none of these pathways, underscoring the need for dedicated mechanistic studies in appropriately characterized patient populations.

## 7. Conclusions

CHIP and TMAO converge on the same NLRP3–IL-1β effector pathway through mechanistically distinct but compatible routes, and the gut microbiome is causally upstream of both. The CHIDT framework proposes that these pathways are not merely co-occurring but mutually reinforcing: CHIP-driven intestinal immune dysregulation plausibly promotes TMA-producing gut ecologies, while elevated TMAO provides an exogenous inflammasome stimulus whose effect is predicted to be amplified in *TET2*-mutant myeloid cells already primed by somatic mutation.

The evidentiary basis for this interaction is circumstantial but mechanistically grounded. The gut–CHIP–inflammation circuit has direct murine support [38]. The weakest element of the framework is the molecular bridge itself. The TMAO→TET2 molecular bridge—transcriptional suppression in VECs via CYTB promoter hypermethylation—is mechanistically precise but established only at supraphysiological concentrations; whether TET2 haploinsufficiency lowers the operative threshold to clinically encountered levels is the central experimental gap.

Scope. The CHIDT hypothesis, in its most evidence-grounded formulation, applies to individuals carrying TET2-CHIP. The extension to DNMT3A-CHIP, to individuals without CHIP, and to populations with physiological-range TMAO concentrations is a plausible but unvalidated generalization that requires targeted experimental work before it can be considered established.

Limitations. Four empirical gaps define the current boundaries of the framework. First, the TMAO→TET2 molecular bridge has been demonstrated only at ~600 μmol/L in cultured VECs; whether the same mechanism operates at the 1–50 μmol/L range encountered in patients with chronic kidney disease or high-choline dietary intake is unknown. Second, supra-additive NLRP3 activation in TET2-haploinsufficient macrophages exposed to pathophysiological TMAO concentrations has not been shown in any published study. Third, whether TET2-CHIP systematically promotes specifically TMA-producing gut dysbiosis in proportion to variant allele fraction has not been measured in humans. Fourth, no cohort has yet co-measured CHIP genotype, gut metagenome TMA-lyase operon abundance, and plasma TMAO in the same individuals at sufficient scale to test the CHIDT hypothesis epidemiologically.

Research priorities. Immediate research priorities are organized into four modules. Module 1 (in vitro): treatment of Tet2+/− and wild-type bone-marrow-derived macrophages with TMAO at 1–50 μmol/L; primary endpoints: NLRP3 assembly (ASC speck formation), caspase-1 cleavage, IL-1β secretion, and 5hmC abundance at the CYTB promoter. Module 2 (animal models): Tet2-CHIP bone marrow chimeric mice (Ldlr^−^/^−^ background) on a high-choline diet (1.2% *w*/*w*) for 12 weeks, with and without DMB; endpoints: plasma TMAO (LC-MS/MS), aortic plaque burden, gut metagenome (cntA/gmhA operon abundance), and 5hmC sequencing in aortic VECs. Module 3 (clinical cohorts): cross-sectional analysis in biobanks with available whole-exome or targeted CHIP sequencing, fasting plasma TMAO, and fecal metagenomics, with adjustment for diet, eGFR, loop diuretic use, statin use, and sex. Module 4 (intervention): Phase II RCT in TET2-CHIP carriers (VAF ≥5%, plasma TMAO ≥6 μmol/L) randomized to colchicine, berberine, or combination; primary endpoints: plasma IL-1β, IL-18, TMAO, and clone size by ddPCR at 6 months.

Immediate research priorities are cross-sectional studies linking CHIP genotype to gut TMA-lyase operon abundance and plasma TMAO; mechanistic in vitro work in *Tet2*-haploinsufficient cells at clinically relevant TMAO concentrations; and murine studies combining *Tet2*-CHIP bone marrow chimeras with high-choline dietary TMAO induction. Population cohorts with concurrent whole-exome sequencing, fecal metagenomics, and metabolomic TMAO quantification will subsequently be needed to determine whether synergistic cardiovascular hazard is detectable at the epidemiological scale. As CHIP genotyping and plasma TMAO measurement become progressively integrated into clinical practice, the opportunity to identify individuals at the intersection of these two risk axes—and to test whether targeting the shared TET2–NLRP3 inflammatory nexus yields benefit beyond single-factor intervention—is a concrete and tractable question for precision cardiovascular prevention.

## Data Availability

The data used to support the findings of this study are included in the article.
